# Differential Effects of IL-2 and IL-6 on the Development of Three Distinct Precursor T-Cell Populations in the Thymus

**DOI:** 10.1155/1990/63249

**Published:** 1990

**Authors:** Naoko Nakano, Hitoshi Kikutani, Tadamitsu Kishimoto

**Affiliations:** Division of Immunology Institute for Molecular and Cellular Biology Osaka University 1-3, Yamada-Oka Suita City Osaka 565 Japan

## Abstract

Three distinct T-cell precursors: bone marrow cells that express low levels of the Thy-1 antigen but no lineage markers (Thy-1-lo/BM); CD4^-^, CD8^-^, and CD3^-^ thymocytes that express low levels of the Thy-1 antigen (Thy-1-lo/Thym); and CD4^-^, CD8^-^, and CD3^-^ thymocytes that express high levels of the Thy-1 antigen and the IL-2 R α chain (Thy-1^+^/ IL2R^+^) were isolated by fluorescence-activated cell sorter (FACS). These three populations expanded with different kinetics in the thymus of irradiated recipient mice after intrathymic transfer. When a high dose of human recombinant IL-2 (r-IL-2) or human recombinant IL-6 (r-IL-6) was administered, r-IL-6 accelerated donor Thy-1^+^/IL2R^+^ to differentiate, whereas r-IL-2 blocked normal differentiation and expansion of donor Thy-1-lo/Thym, but did not show any significant effect on donor Thy-1^+^/IL2R^+^. Neither r-IL-2 nor r-IL-6 worked directly on donor Thy-1-lo/BM in this transfer system.


*Developmental Immunology*, 1990, Vol. 1, pp. 77–84
Reprints available directly from the publisher
Photocopying permitted by license only

© 1990 Harwood Academic Publishers GmbH
Printed in the United Kingdom

### Differential Effects of IL-2 and IL-6 on the Development of Three Distinct Precursor T-Cell Populations in the Thymus

NAOKO NAKANO, HITOSHI KIKUTANI, and TADAMITSU KISHIMOTO

*Division of Immunology, Institute for Molecular and Cellular Biology, Osaka University, 1-3, Yamada-Oka, Suita City, Osaka 565, Japan*

Three distinct T-cell precursors: bone marrow cells that express low levels of the Thy-1 antigen but no lineage markers (Thy-1-lo/BM); $CD4^-$, $CD8^-$, and $CD3^-$ thymocytes that express low levels of the Thy-1 antigen (Thy-1-lo/Thym); and $CD4^-$, $CD8^-$, and $CD3^-$ thymocytes that express high levels of the Thy-1 antigen and the IL-2 R $\alpha$ chain ($Thy\text{-}1^+/IL2R^+$) were isolated by fluorescence-activated cell sorter (FACS). These three populations expanded with different kinetics in the thymus of irradiated recipient mice after intrathymic transfer. When a high dose of human recombinant IL-2 (r-IL-2) or human recombinant IL-6 (r-IL-6) was administered, r-IL-6 accelerated donor $Thy\text{-}1^+/IL2R^+$ to differentiate, whereas r-IL-2 blocked normal differentiation and expansion of donor Thy-1-lo/Thym, but did not show any significant effect on donor $Thy\text{-}1^+/IL2R^+$. Neither r-IL-2 nor r-IL-6 worked directly on donor Thy-1-lo/BM in this transfer system.

KEYWORDS: IL-2, IL-6, T-cell progenitor, thymic development.

### INTRODUCTION

Development of T cells in the thymus can be divided into two major processes: early T-cell development before the acquirement of T-cell-receptor (TCR) expression and TCR-mediated maturation of T cells such as "positive" and "negative" selections. Early events of TCR-independent T-cell development are further characterized by (1) commitment of stem cells to T-cell lineage, presumably in the bone marrow, (2) migration of precursor T cells into the thymus, and (3) rearrangement of TCR genes before expressing CD4/CD8 differentiation antigens. All of these processes are essential to provide progenitor cells that are to be selected within the thymus and eventually cover all the T-cell repertoire required.

Recently, a number of studies have been carried out to fractionate $CD4^-/CD8^-$ (double-negative) thymocytes on the basis of cell-surface phenotypes, such as Thy-1, Ly-1, Pgp-1, interleukin-2 receptor (IL-2R), J11d, and B2A2 (Ceredig et al., 1985; Fowlkes et al., 1985; Raulet, 1985; Husman et al., 1988; Scollay and Shortman, 1985; Rearse et al., 1989). Previously, we identified the most immature double-negative thymocyte population that lacks IL-2R and CD3 expression, but expresses low levels of the Thy-1 antigen (Nakano et al., 1987). These cells reconstituted T cells in irradiated host mice when they were transferred intravenously. There is also a distinct population of double-negative thymocytes that expresses high levels of Thy-1 and IL-2R, and is known to give rise to mature thymocytes efficiently when they are injected into the thymus directly (Shimonkevitz et al., 1987). It has been demonstrated that purified stem cells from the bone marrow have a potential to give rise to T cells when they are injected intrathymically. Such cells lack differentiation markers such as T cells, B cells, macrophages, and granulocytes, but express the Sca-1 antigen and low levels of the Thy-1 antigen (Spangrude et al., 1988). Although precursors in the thymus and in the bone marrow are apparently different, it is still obscure in what stage of differentiation they are and what kind of growth factors are required for their expansion and differentiation.

IL-2 induces proliferation of activated T cells. However, it is not clear how IL-2 is involved in T-cell ontogeny, although a certain population of thymocytes are known to express the $\alpha$ chain of IL-2R. Recently, one of the pleiotropic factors, interleukin-6 (IL-6) (Hirano and Kishimoto, 1990), also has been reported to work as a T-cell growth factor (Le et al., 1988; Uttenhove et al., 1988), and it is possible that IL-6 plays a role in thymocyte development.

In an attempt to understand the differentiation process of precursor T cells, we took advantage of intrathymic injection (Goldschneider et al., 1986), combining with the three-color fluorescence-activated cell sorter (FACS) to isolate distinct T-cell precursors and to chase the fate of only donor-derived thymocytes in the host thymus. Effects of IL-2 and IL-6 on the development of T cells were also studied in this experimental system by administration of a relatively large amount of human recombinant IL-2 (r-IL-2) or human recombinant IL-6 (r-IL-6) to recipient mice that had been injected with sorted precursor T cells.

### RESULTS

### Capacity of Expansion and the Stage of Development of Precursor T Cells

On the basis of Thy-1 and IL-2-R expression, double-negative and CD3-negative C3H/HeN thymocytes were subfractionated into three populations, IL-2-R$^+$ and IL-2-R$^-$ thymocytes (Thy-1$^+$/IL2R$^+$ and Thy-1$^+$/IL2R$^-$, respectively), both of which express high levels of the Thy-1 antigen, and thymocytes with low levels of the Thy-1 antigen (Thy-1-lo/Thym) (Nakano et al., 1987). Bone marrow cells expressing low levels of the Thy-1 antigen (Thy-1-lo/BM), which have been demonstrated to give rise to T cells as well as all lineages of hematopoietic cells (Muller-Sieburg et al., 1986), were also sorted from C3H/HeN bone marrow cells as another source of precursor T cells. Sorted precursor T-cell populations were intrathymically injected into sublethally irradiated AKR/J recipients and examined for their ability to reconstitute the recipient thymuses.

T cells derived from injected precursor cells were chased by staining the Thy-1.2 antigen, which allows distinguishing T cells of donor origin from those of host AKR/J (Thy-1.1), and Fig. 1A shows kinetics of expansion of donor-derived T cells detected as Thy-1.2$^+$. When $1\times10^6$ Thy-1$^+$/IL2R$^-$ were injected, cells detectable as Thy-1.2$^+$ tapered off within a few days and did not expand in the thymuses of irradiated recipients. In the recipient thymuses injected with $2\times10^5$ Thy-1$^+$/IL2R$^+$ cells,

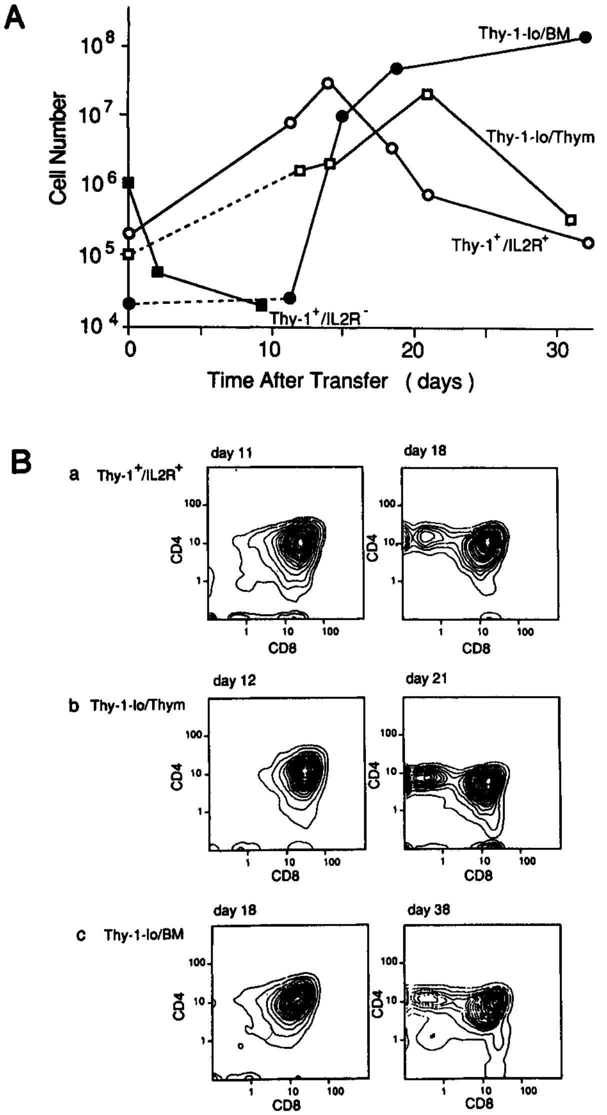

FIGURE 1. (A) Expansion of donor precursor T cells in the thymus after intrathymic injection. Either $2\times10^4$ Thy-1-lo/BM (closed circle), $1\times10^5$ Thy-1-lo/Thym (open square), $2\times10^5$ Thy-1$^+$/IL2R$^+$ (open circle), or $1\times10^6$ Thy-1$^+$/IL2R$^-$ (closed square) from C3H/HeN (Thy-1.2) was injected intrathymically into sublethally (690 rad) irradiated AKR/J (Thy-1.1) mice. Thymocytes prepared from the recipient mice were analyzed by FACS 440 on the day indicated. Mean values of numbers of Thy-1.2$^+$ cells in two to three different transfers were shown. Each cell number was calculated from the total number of thymocytes and the percentage of Thy-1.2-positive cells. (B) Expression of CD4 and CD8 on donor type (Thy-1.2$^+$) thymocytes in recipient thymuses injected with either (a) Thy-1$^+$/IL2R$^+$, (b) Thy-1-lo/Thym, or (c) Thy-1-lo/BM. Thymocytes were prepared at indicated time points after the transfer of each precursor population, and stained with FITC-anti-CD8, PE-anti-CD4, and PC-anti-Thy-1.2 except for the right panel in (C), where PC-anti-CD4 and PE-anti-Thy-1.2 were used with FITC-anti-CD8.

Thy-1.2$^+$ donor-derived cells expanded one hundredfold within 10–14 days and decreased thereafter. T cells derived from this precursor population were mostly CD4$^+$/CD8$^+$ on day 11, and gradually shifted toward CD4$^+$/CD8$^-$ and CD4$^-$/CD8$^+$ on day 18, Fig. 1B(*a*). A delay in expansion of donor-derived thymocytes was observed when 1×10$^5$ Thy-1-lo/Thym was injected. Although cells detected as Thy-1.2$^+$ expressed mostly both CD4 and CD8 on day 12, their cell number was only tenfold higher than that of injected cells, Fig. 1A and Fig. 1B(*b*). They continued to expand and reached the maximum level on day 21 with one hundredfold expansion. As shown in Fig. 1B(*b*), the differentiated cells such as single positive cells were observed on day 21. The delay of progression and expansion indicates that Thy-1-lo/Thym is a more immature population.

It took much longer for Thy-1-lo/BM to differentiate into double positive. Cells detected as Thy-1.2$^+$ were still double negative 11 days after injection of 2×10$^4$ Thy-1-lo/BM (data not shown). However, Thy-1.2$^+$ cells were composed of double-positive cells on day 18, as shown in Fig. 1B(*c*). Thy-1-lo/BM-derived cells increased sharply after 14 days and occupied almost the whole thymus one month later. In contrast to Thy-1$^+$/IL2R$^+$ and Thy-1-lo/Thym, which could transiently reconstitute the thymus, Thy-1-lo/BM had a potential to expand 10 thousandfold (Fig. 1A) and this state continued over 2 months. When cells were analyzed 38 days later, half of the thymocytes were still of donor type (Thy-1.2$^+$) and their phenotypes shown in Fig. 1B(*c*) were similar to those in normal mice.

In this system, the kinetics and the extent of expansion and differentiation appear to indicate the differentiation stage of precursor T cells; Thy-1$^+$/IL2R$^-$ is the most differentiated population because of its incapability of expansion, and Thy-1$^+$/IL2R$^+$ is more differentiated than Thy-1-lo/Thym, which was indicated by its delayed kinetics of thymus reconstitution. Thy-1-lo/BM differs completely from Thy-1-lo/Thym in terms of capacity of expansion and duration of reconstitution, although its phenotype is similar to Thy-1-lo/Thym.

### IL-6 Promotes Thy-1$^+$/IL2R$^+$ to Differentiate into CD4$^+$/CD8$^+$ and CD4$^+$/CD8$^-$

We employed this transfer system to investigate the effects of r-IL-2 and r-IL-6 on the reconstitution of the thymus by three T-progenitor populations: Thy-1$^+$/IL2R$^+$, Thy-1-lo/Thym, and Thy-1-lo/BM, all of which were capable to expand in the thymus, as described before. First, irradiated AKR/J recipients were intrathymically transferred with Thy-1$^+$/IL2R$^+$ C3H/HeN cells and then injected with saline, 10 $\mu$g of r-IL-2, or 10 $\mu$g of r-IL-6 every day. By gating on donor-derived Thy-1.2 positive cells, the expression of CD4 and CD8 on the donor-derived thymocytes was analyzed on day 11. As shown in Fig. 2A, surface phenotypes of donor-derived cells were mostly CD4$^+$/CD8$^+$ at this point. The relative proportion of CD4$^-$/CD8$^-$ cells in r-IL-6-treated mice was lower than in the control, Fig. 2A(*c*). The absolute number of donor-derived cells in r-IL-6-treated recipient thymus was found to be several times higher than that in the control. This difference was much evident in CD4$^+$/CD8$^+$ and CD4$^+$/CD8$^-$ populations (Table 1, Exp. 1 and 2). On day 14, on the contrary, donor-derived cells were less in r-IL-6-treated mice than in control mice (Table 1, Exp. 3). This result suggests that r-IL-6 may accelerate the differentiation from Thy-1$^+$/IL2R$^+$ cells to CD4$^+$/CD8$^+$ cells. A little or no increase of donor-derived cells was observed in r-IL-2-treated mice in Table 1, althrough these cells expressed the $\alpha$ chain of IL-2R.

### IL-2 Blocks Normal Differentiation of Thy-1-lo/Thym

Thy-1-lo/Thym was similarly injected into the thymus of irradiated recipients and 10 $\mu$g of either r-IL-2 or r-IL-6 were administered every day. On day 14, mice were sacrificed and CD4 and CD8 expressions of Thy-1.2$^+$ donor-derived thymocytes were analyzed. Although phenotypes of thymocytes in r-IL-6-treated recipients were similar to those in saline-treated controls, Fig. 2B(*a*) and (*c*), more progeny derived from Thy-1-lo/Thym was detected in r-IL-6-treated mice than in controls on day 14 (Table 2, Exp. 1), then less on day 18 (Table 2, Exp. 3). We suppose Thy-1-lo/Thym passes though the Thy-1$^+$/IL2R$^+$ stage (data not shown), so that the effect obtained by r-IL-6 on Thy-1-lo/Thym may have been exerted on that stage.

In contrast, r-IL-2 treatment caused a dramatic effect on the expansion and differentiation of Thy-1-lo/Thym. Their phenotypes turned out more widespread. The relative proportions of single-positive cells and double-negative cells increased in r-IL2-treated mice, whereas that of double-positive cells decreased, as shown in Fig. 2B(*b*). Interestingly, actual cell numbers of all populations, particularly

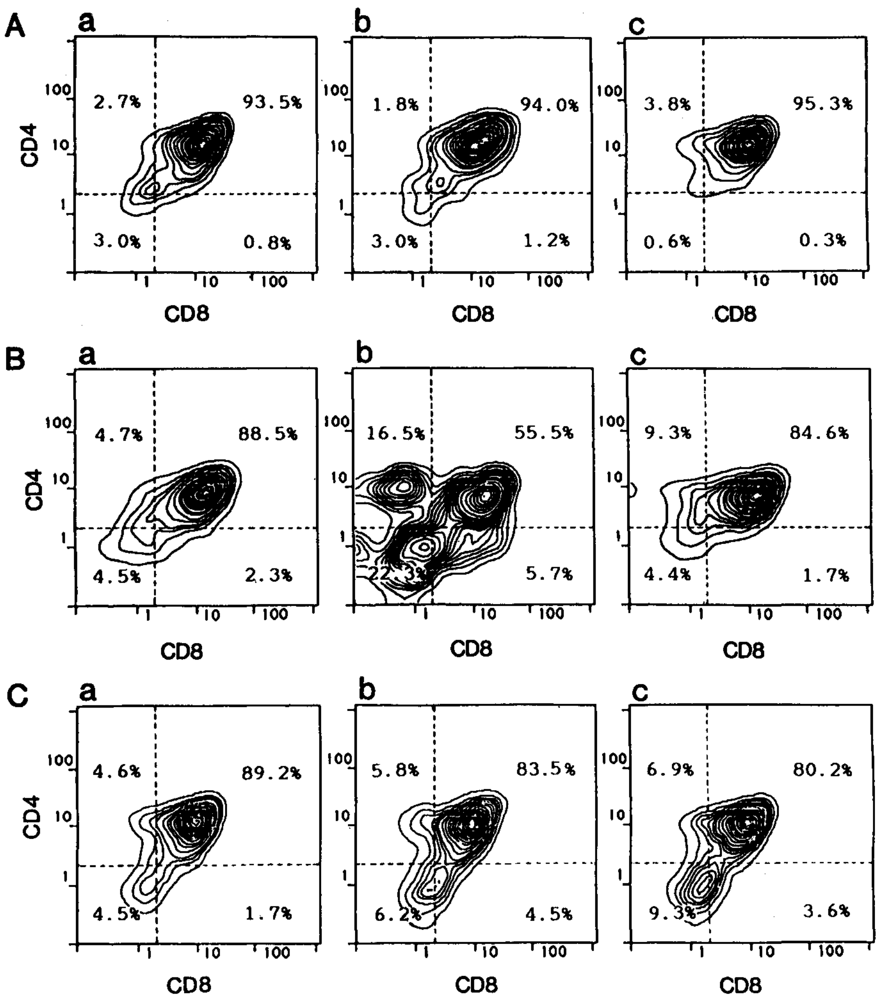

FIGURE 2. Effects of r-IL-2 and r-IL-6 on the development of donor-derived thymocytes. (A) 1×10$^5$ Thy-1$^+$/IL2R$^+$, (B) 1×10$^5$ Thy-1-lo/Thym, or (C) 1×10$^4$ Thy-1-lo/BM isolated from C3H/HeN mice were transferred intrathymically into irradiated AKR/J mice as mentioned in Figure 1. Either (a) saline, (b) 10 μg r-IL-2, or (c) 10 μg r-IL-6 was administered daily. Thymocytes were analyzed on (A) day 11, (B) day 14, or (C) day 15, staining with FITC-anti-CD8, PC-anti-CD4, and PE-anti-Thy-1.2. Profiles of cells gated positively on Thy-1.2 were shown.

TABLE 1
The Effect of r-IL-2 and r-IL-6 on the Reconstitution of the Thymus with Thy-1$^+$/IL2R$^+$ Cells[a]

| Exp. | Days after transfer | Treated with | Donor type cells in the thymus (×10$^{-5}$) | | | | |
|---|---|---|---|---|---|---|---|
| | | | Total | CD4$^-$CD8$^-$ | CD4$^+$CD8$^+$ | CD4$^+$CD8$^-$ | CD4$^-$CD8$^+$ |
| 1 | 11 | Saline | 7.3 | 0.21 | 6.8 | 0.20 | 0.059 |
| | | r-IL-2 | 6.3 | 0.19 | 5.9 | 0.11 | 0.075 |
| | | r-IL-6 | 46 | 0.27 | 44 | 1.7 | 0.15 |
| 2 | 11 | Saline | 3.1 | 0.48 | 2.5 | 0.11 | 0.012 |
| | | r-IL-2 | 13 | 0.49 | 12 | 0.46 | 0.047 |
| | | r-IL-6 | 34 | 0.65 | 32 | 1.3 | 0.038 |
| 3 | 14 | Saline | 190 | 0.57 | 172 | 17 | 1.2 |
| | | r-IL-2 | 192 | 0.50 | 177 | 13 | 1.0 |
| | | r-IL-6 | 81 | 0.26 | 71 | 8.5 | 0.85 |

[a]1×10$^5$ Thy-1$^+$/IL2R$^+$ cells sorted from C3H/HeN (Thy-1.2) were injected intrathymically into 690-rad irradiated AKR/J (Thy-1.1) mice. Human r-IL-2 (10 μg), r-IL-6 (10 μg), or saline was administered to the recipients once a day until the day before the analysis. Numbers of donor-derived cells (Thy-1.2$^+$) were calculated from the number of total thymocytes in the recipient mouse and the data of the three-color FACS analysis.

TABLE 2
The Effect of r-IL-2 and r-IL-6 on the Reconstitution of the Thymus with Thy-1-lo/Thym Cells[a]

| Exp. | Days after transfer | Treated with | Donor type cells in the thymus ( $\times 10^{-5}$) Total | $CD4^-CD8^-$ | $CD4^+CD8^+$ | $CD4^+CD8^-$ | $CD4^-CD8^+$ |
|---|---|---|---|---|---|---|---|
| 1 | 14 | Saline | 21 | 0.97 | 19 | 1.0 | 0.50 |
| | | r-IL-2 | 2.7 | 0.59 | 1.5 | 0.44 | 0.16 |
| | | r-IL-6 | 70 | 3.1 | 59 | 6.5 | 1.2 |
| 2 | 14 | Saline | 23 | 1.1 | 19 | 1.3 | 0.31 |
| | | r-IL-2 | 0.86 | 0.22 | 0.40 | 0.044 | 0.20 |
| | | r-IL-6 | 29 | 1.3 | 25 | 2.2 | 0.34 |
| 3 | 18 | Saline | 23 | 1.1 | 20 | 1.4 | 0.31 |
| | | r-IL-2 | 0.33 | 0.17 | 0.13 | 0.013 | 0.018 |
| | | r-IL-6 | 4.4 | 1.0 | 2.7 | 0.40 | 0.29 |

[a] $1 \times 10^5$ Thy-1-lo/Thym cells from C3H/HeN were injected intrathymically into irradiated AKR/J (Thy-1.1) recipients. The other procedures were described in the footnote for Table 1.

TABLE 3
The Effect of r-IL-2 and r-IL-6 on the Reconstitution of the Thymus with Thy-1-lo/BM Cells[a]

| Exp. | Days after transfer | Treated with | Donor Type cells in the thymus ( $\times 10^{-5}$) Total | $CD4^-CD8^-$ | $CD4^+CD8^+$ | $CD4^+CD8^-$ | $CD4^-CD8^+$ |
|---|---|---|---|---|---|---|---|
| 1 | 15 | Saline | 104 | 6.1 | 89 | 6.2 | 2.3 |
| | | r-IL-2 | 48 | 3.0 | 40 | 2.8 | 2.2 |
| | | r-IL-6 | 173 | 16 | 139 | 12 | 6.2 |
| 2 | 15 | Saline | 107 | 3.2 | 94 | 8.8 | 0.66 |
| | | r-IL-2 | 37 | 0.32 | 34 | 2.3 | 0.11 |
| | | r-IL-6 | 108 | 1.6 | 96 | 9.6 | 0.49 |

[a] $1 \times 10^4$ Thy-1-lo/BM cells were transferred intrathymically into irradiated recipient mice. The other procedures were described in the footnote for Table 1.

$CD4^+/CD8^+$ and $CD4^+/CD8^-$, were reduced extremely in r-IL-2-treated mice (Table 2). This result indicates that r-IL-2 interferes with the expansion and differentiation of Thy-1-lo/Thym.

### Thy-1-lo/BM Cells as Precursor T Cells Are Not Sensitive to Either r-IL-2 or r-IL-6

Irradiated AKR/J mice were injected intrathymically with $1 \times 10^4$ Thy-1-lo/BM cells and administered with r-IL-2, r-IL-6, or saline every day. Phenotypes of donor-derived cells in the recipient thymuses were analyzed as in other transfers. On day 15, as shown in Fig. 2C(*a*), the main population of transferred cells was double positive and phenotypic patterns of thymocytes were almost identical among three treatments (Fig. 2C). We did not find any clear effect of r-IL-6 on the expansion of progeny of Thy-1-lo/BM cells, as shown in Table 3. Some reduction of cell numbers in r-IL-2-treated mice was observed, possibly because Thy-1-lo/BM differentiated into the stage of Thy-1-lo/Thym, where r-IL-2 blocked the differentiation.

### DISCUSSION

We isolated three subpopulations of double-negative and CD3-negative thymocytes as well as T progenitors from bone marrow cells and examined their growth and differentiation in the thymus. $Thy\text{-}1^+/IL2R^-$, which had been previously shown to directly differentiate into double-positive cells in an in vitro culture (Nakano et al., 1987), failed to expand in the thymus (Fig. 1A) and was considered as the most differentiated population in double-negative/$CD3^-$ thymocytes. Both $Thy\text{-}1^+/IL2R^+$ and Thy-1-lo/Thym populations could expand and transiently reconstitute the recipient thymuses. However, the kinetics of the expansion of these two populations was different. The peak of expansion of $Thy\text{-}1^+/IL2R^+$ was observed on day 14, whereas the number of progeny of Thy-1-lo/Thym population was maximum on day 21 and then decreased. This observation suggests that Thy-1-lo/Thym may be more immature than Thy-$1^+$/IL2$R^+$ and coincides with our previous results obtained by intravenous transfer and in vitro culture (Nakano et al., 1987). In the previous experiment (Nakano et al., 1987), Thy-$1^+$/IL2$R^+$ cells failed to reconstitute the thymus when transferred intravenously. This may be due to the difference of the route of the transfer or the timing of analysis. Similar subfractionations of immature thymocytes on the basis of the IL-2R $\alpha$-chain expression have been reported. Shimonkevitz et al. (1987) divided double-negative thymocytes into two subpopulations, IL-2$R^+$ and IL-2$R^-$ cells, and showed that IL-2$R^+$ cells had more capacity to reconstitute the thymus by using intrathymic injection. Recently, Rearse et al. (1989) fractionated double-negative thymocytes by using two markers, Pgp-1 and IL-2R, and demonstrated that the differentiation sequence of immature thymocytes was Pgp-$1^+$/IL2$R^-$$\rightarrow$Pgp-$1^+$/IL2$R^+$$\rightarrow$Pgp-$1^-$/IL2$R^-$. The present study showed that the IL-2$R^+$ population in double-negative thymocytes was at an intermediate stage between Thy-1-lo/Thym and Thy-$1^+$/IL2$R^-$. If we assume that Thy-1-lo/Thym cells express the Pgp-1 antigen, their results may be consistent with ours.

The reconstitution by Thy-1-lo/BM cells lasted for at least 2 months (data not shown) in contrast to the transient reconstitution by Thy-1-lo/Thym and Thy-$1^+$/IL2$R^+$. A similar observation was obtained by Katsura et al. (1988) from their intrathymic transfer experiments using unfractionated cells from thymus, bone marrow (BM), and spleen as a source of T-cell progenitors. These findings suggest that precursors in the thymus have a poor self-renewal activity compared to those in BM. However, it is likely that Thy-1-lo/BM still includes hematopoietic progenitors of different stages such as Thy-1-lo/Sca-$1^+$ pluripotent stem cells (Spangrude et al., 1988). Therefore, there are two possibilities: (1) pluripotent hematopoietic stem cells, which have self-renewal activity, may participate in the expansion and differentiation exclusively to T-cell lineage in the thymus, or (2) a certain progenitor population in Thy-1-lo/BM, which is already committed to T lineage, but more immature than Thy-1-lo/Thym, may be the cells responsible for the reconstitution of the thymus. In fact, it was reported that Thy-1-lo/Sca-$1^+$ pluripotent stem cells could reconstitute the thymus when they were transferred intrathymically (Spangrude et al., 1988). This favors the former possibility. Whichever is the case, Thy-1-lo/BM differs completely from Thy-1-lo/Thym in terms of reconstitution of the thymus.

Progenitor T cells homed in the thymus proliferate and differentiate under the influence of the microenvironment in the thymus. The intrathymic transfer system is thus useful for the analysis of the effect of growth factors on precursor cells developing in concert with the thymic environment. This study demonstrated that r-IL-2 and r-IL-6 exerted their effect on different stages of precursor T cells. r-IL-6 was found to work preferentially on Thy-$1^+$/IL2$R^+$, resulting in the acceleration of thymus reconstitution by this population. Previously, r-IL-6 was shown to enhance hematopoietic activity of bone marrow cells (Ikebuchi et al., 1987; Koike et al., 1988). In our system, however, r-IL-6 showed little effect on the reconstitution with Thy-1-lo/BM. One may argue that r-IL-6 should have some effect on the expansion and differentiation of Thy-1-lo/Thym or Thy-1-lo/BM, since these cells pass through the Thy-$1^+$/IL2$R^+$ stage on the process of differentiation. In fact, the treatment with r-IL-6 sometimes enhanced the expansion of Thy-1-lo/Thym; we observed 2 to 3 times more progeny of Thy-1-lo/Thym in r-IL-6-treated mouse in Exp. 1 of Table 2.

The administration of r-IL-2 to the recipient mice that were transferred with Thy-1-lo/Thym caused a dramatic reduction of the donor-derived cells both on day 14 and day 18. There are two possibilities: (1) IL-2 blocked the growth and differentiation of Thy-1-lo/Thym or (2) r-IL-2 accelerated the differentiation of Thy-1-lo/Thym. If the latter is the case, we should have seen Thy-1.$2^+$ T cells in the periphery. However, we did not detect those cells in lymph nodes (data not shown). Although the absolute number of donor-derived thymocytes decreased, the relative ratio of CD$4^+$/CD$8^-$, CD$4^-$/CD$8^+$, and CD$4^-$/CD$8^-$ cells derived from transferred Thy-1-lo/Thym increased in the thymus of r-IL-2-treated recipients. There are two possibilities: (1) the dose of r-IL-2 was not sufficient for the inhibition of all Thy-1-lo/Thym, resulting in the development of some cells normally, or (2) Thy-1-lo/Thym may be still heterogeneous with regards to the sensitivity to r-IL-2. Interestingly, Thy-1-lo/Thym cells do not express the IL-2R $\alpha$ chain. The inhibitory effect of IL-2 might be exerted through the IL-2R $\beta$ chain. Since NK cells were shown to express the $\beta$ chain of IL-2R (Shimonkevitz et al., 1987), it may be reasonable to assume its presence in Thy-1-lo/Thym. In vitro isolated IL-2R$^+$ double-negative thymocytes were reported not to be responsive to IL-2 (von Boehmer et al., 1985), but responsive with additional stimuli like PMA plus ionomycine (Lowenthal et al., 1986). Jenkinson et al. (1987) and Tentori et al. (1988) showed that anti-IL-2R $\alpha$-chain antibodies blocked the generation of mature T cells in thymic organ culture as well as in vivo, demonstrating an importance of IL-2–IL-2R interaction in thymocyte differentiation. Effects of IL-2 on the development of precursor T cells have been suggested (Toribio et al., 1988). However, in the present study, exogenously administered IL-2 did not show much effect on this distinct Thy-1$^+$/IL2R$^+$ stage, although a small increase of donor-derived cells was occasionally observed (Table 1. Exp. 2). The controversy might be explained by assuming that endogenously generated IL-2 might be sufficient for the development of Thy-1$^+$/IL2R$^+$ cells. The antibodies against the human $\beta$ chain of IL-2R as well as the human IL-6R are now available (Hirata et al., 1989; Takeshita et al., 1989). Therefore, reagents against murine counterparts of them will enable us to do more detailed studies on the role of these growth factors on T-cell maturation.

### MATERIALS AND METHODS

### Antibodies

Monoclonal antibodies (mAbs) were purified from culture supernatants of hybridomas 53-6.7 (anti-CD8) (Ledbetter et al., 1982), GK-1.5 (anti-CD4) (Dialynas et al., 1983), 53-7.3 (anti-CD5), MAR18.5 (mouse anti-rat Ig$\kappa$), 30H12 (anti-Thy1.2) (Ledbetter et al., 1979), PC61 (anti-IL-2R) (Ceredig et al., 1985), M1/70 (anti-Mac-1) (Springer et al., 1979) and 53-10.1 (Ledbetter et al., 1979) as previously described (Hardy, 1986). Fluorescein (FITC), biotin (bi) or phycobiliprotein labeling has been described (Hardy, 1986).

### Cell Preparation and Sorting

Double-negative thymocytes were prepared from 3–5 week C3H/HeN mice, as previously described (Muller-Sieburg et al., 1986) by treating with a cocktail of antithymocyte antibodies (53-6.7, GK1.5, 53-7.3), and with MAR18.5 as a facilitating reagent. After treatment with rabbit complement (low-tox-M, Cedarlane, Ontario, Canada), approximately 90% pure double-negative thymocytes were obtained. Then these cells were stained with FITC-labeled anti-CD4 and FITC-anti-CD8, bi-anti-IL-2R and phycocyanin (PC)-anti-Thy1.2, followed by phycoerythrin (PE)-avidin for IL-2R$^+$ cell sorting. All double-negative IL-2R$^+$ thymocytes were Thy-1$^+$, and 30–40% of double-negative thymocytes belonged to this fraction. This fraction was used as Thy-1$^+$/IL2R$^+$. To obtain Thy-1-lo/Thym, double-negative thymocytes enriched as described before were stained with FITC-anti-CD4, FITC-anti-CD8, PE-anti-Thy.1.2, and PC-anti-CD3, and cells expressing low levels of Thy-1.2 but lacking CD3 expression among double-negative thymocytes were sorted. Bone marrow cells were prepared from femurs and tibias of 3–5-week-old C3H/HeN mice. Hemolyzed bone marrow cells were stained with FITC-anti-B220, FITC-anti-Mac-1, FITC-53-10.1, FITC-anti-CD4, FITC-anti-CD8, and PC-anti-Thy-1.2. Cells expressing low levels of Thy-1 and not detectable for FITC stainings were sorted. This population was approximately 0.5% of total bone marrow cells and used as Thy-1-lo/BM. All the sorting and analysis were done with FACS 440 dual-laser dye-laser cell sorter (Becton Dickinson, Mountain View, CA).

### Precursor-Cell Transfer and Analysis

Sorted precursor populations from C3H/HeN mice (Thy-1.2) were resuspended in Hanks' balanced salt solution and injected intrathymically into 7–10 week-old female AKR/J mice (Thy-1.1) that had been $\gamma$-irradiated (690 rad). Methods of injection were described previously (Goldschneider et al., 1986). For the analysis of donor-derived cells, thymocytes or lymph node cells were routinely stained with FITC-anti-CD8, PC-anti-CD4, and PE-anti-Thy-1.2, or with another combination using FITC-anti-CD8, PE-anti-CD4, and PC-anti-Thy-1.2, and analyzed with FACS 440. The expression of CD4 and CD8 in donor-derived cells was examined by gating on cells positive for donor Thy-1 marker(Thy-1.2$^+$).

### Interleukin Treatment

Human r-IL-6 has been described (Hirano et al., 1986). Human r-IL-2 was a kind gift of Ajinomoto Co. Ltd., Tokyo. Ten $\mu$g of either r-IL-2 or r-IL-6 in 200 $\mu$l saline were injected intravenously immediately after cell transfer. Following days, the same amount of interleukins was injected into the peritoneal cavity once a day. Control mice were injected with the same volume of saline.

### ACKNOWLEDGMENTS

We thank Dr. Y. Katsura, Kyoto University, for the instruction of intrathymic injection, and Ms. K. Kubota and Ms. M. Harayama for their assistance.

*(Received April 15, 1990)*

*(Accepted April 20, 1990)*

### REFERENCES

Ceredig R., Lowenthal J.W., and MacDonald H.R. (1985). Expression of interleukin-2 receptors as a differentiation marker on intrathymic stem cells. Nature **314,** 98–100.

Coffman R. (1983). Surface antigen expression and immunoglobulin gene rearrangement during mouse pre-B cell development. Immunol. Rev. **69,** 5–23.

Dialynas D.P., Wilde D.B., Marrack P., Pierres A., Wall K.A., Havran W., Otten G., Loken M.R., Pierres M., Kappler J., and Fitch F.W. (1983). Characterization of the murine antigenic determinant, designated L3T4a, recognized by monoclonal antibody GK1.5: expression of L3T4a by functional T cell clones appears to correlate primarily with class II MHC antigen-reactivity. Immunol. Rev. **74,** 29–56.

Fowlkes B.J., Edison L., Mathieson B.J., and Chused T.M. (1985). Early T lymphocytes: Differentiation in vivo of adult intrathymic precursor cells. J. Exp. Med. **162,** 802–822.

Goldschneider I., Komschlies K.L., and Greiner D.L. (1986). Studies of thymocytopoiesis in rat and mice. I. Kinetics of appearance of thymocytes precursors. J. Exp. Med. **163,** 1–17.

Hardy R.R. (1986). Purification and coupling of fluorescent proteins for use in flow cytometry. *In:* Handbook of experimental immunology, vol. 1, Weir, D.M., Ed. (Boston: Blackwell Scientific Publications), pp. 13.1–13.13.

Hirano T., and Kishimoto T. (1990). Interleukin-6 *In:* Handbook of experimental pharmacology: Peptide growth factors and their receptors, vol. 3, Sporn M.M., and Roberts A.B., Eds. (Berlin: Springer-Verlag), pp. 663–665.

Hirano T., Yasukawa K., Harada H., Taga T., Watanabe Y., Matsuda T., Kashiwamura S., Nakajima K., Koyama K., Iwamatsu S., Sakiyama F., Matsui M., Takahara Y., Taniguchi T., and Kishimoto T. (1986). Complemental DNA for a novel human interleukin (BSF-2) that induces B lymphocytes to produce immunoglobulin. Nature **324,** 73–76.

Hirata Y., Taga T., Hibi M., Nakano N., Hirano T., and Kishimoto T. (1989). Characterization of IL-6 receptor expression by monoclonal and polyclonal antibodies. J. Immunol. **143,** 2900–2906.

Husmann L.A., Shimonkevitz R.P., Crispe I.N., and Bevan, M.J. (1988). Thymocyte subpopulations during early fetal development in the Balb/c mouse. J. Immunol. **141,** 736–740.

Ikebuchi K., Wong G., Clark S.C., Ihle J.N., Hirai Y., and Ogawa M. (1987). Interleukin-6 enhancement of interleukin-3-dependent proliferation of multipotential hemopoietic progenitors. Proc. Natl. Acad. Sci. USA **84,** 9035–9039.

Jenkinson E.J., Kingston R., and Owen J.J.T. (1987). Importance of IL-2 receptors in intra-thymic generation of cells expressing T-cell receptors. Nature **329,** 160–162.

Katsura Y., Kina T., Takaoki Y., and Nishikawa S. (1988). Quantification of the progenitors for thymic T cells in various organs. Eur. J. Immunol. **18,** 889–895.

Koike K., Nakahata T., Takagi M., Kobayashi T., Ishiguro A., Tsuji K., Naganuma M., Okano A., Akiyama Y., and Akabane T. (1988). Synergism of BSF-2/interleukin 6 and interleukin 3 on development of multipotential hemopoietic progenitors in serum-free culture. J. Exp. Med. **168,** 879–890.

Le J., Fredrickson G., Reis L.F.L., Diamantstein T., Hirano T., Kishimoto T., and Vilcek J. (1988). Interleukin-2-dependent and interleukin-2-independent pathways of regulation of thymocyte function by interleukin-6. Proc. Natl. Acad. Sci. USA **85,** 8643–8647.

Ledbetter J.A., and Herzenberg L.A. (1979). Xenogeneic monoclonal antibodies to mouse lymphoid differentiation antigens. Immunol. Rev. **47,** 63–90.

Ledbetter J.A., and Seaman W.E. (1982). The Lyt-2, Lyt-3 macromolecules: Structural and functional studies. Immunol. Rev. **68,** 197–218.

Leo O., Foo M., Sachs D.H., Samelson L.E., and Bluestone, J.A. (1987). Identification of a monoclonal antibody specific for a murine T3 polypeptide. Proc. Natl. Acad. Sci. USA **84,** 1374–1378.

Lowenthal J.W., Howe R.C., Ceredig R., and MacDonald R. (1986). Functional status of interleukin 2 receptors expressed by immature (Lyt-2$^-$/L3T4$^-$) thymocytes. J. Immunol. **137,** 2579–2584.

Muller-Sieburg C.M., Whitlock C.A., and Weissman I.L. (1986). Isolation of two early B cell and a clonogenic Thy-1-lo hematopoietic stem cell. Cell **44,** 653–662.

Nakano N., Hardy R.R., and Kishimoto T. (1987). Identification of intrathymic T progenitor cells by expression of Thy-1, IL-2 receptor and CD3. Eur. J. Immunol. **17,** 1567–1571.

Raulet D.H. (1985). Expression and function of interleukin-2 receptors on immature thymocytes. Nature **314,** 101–103.

Rearse M., Wu L., Egerton M., Wilson A., Shortman K., and Scollay R. (1989). A murine early thymocyte developmental sequence is marked by transient expression of the interleukin 2 receptor. Proc. Natl. Acad. Sci. USA **86,** 1614–1618.

Scollay R., and Shortman K. (1985). Identification of early stages of T lymphocyte development in the thymus cortex and medulla. J. Immunol. **134,** 3632–3642.

Shimonkevitz R.P., Husmann L.A., Bevan M.J., and Crispe, I.N. (1987). Transient expression of IL-2 receptor precedes the differentiation of immature thymocytes. Nature **329,** 157–159.

Siegal J., Sharon P.M., Smith P.L., and Leonard W.J. (1987). Role in mediating signals for LAK, NK and proliferative activities. Science **238,** 75–78.

Spangrude G.J., Heimfeld S., and Weissman I.L. (1988). Purification and characterization of mouse hematopoietic stem cells. Science **241,** 58–62.

Springer T., Galfre G., Secher D.S., and Milstein C. (1979). Mac-1: A macrophage differentiation antigen identified by monoclonal antibody. Eur. J. Immunol. **9,** 301–306.

Takeshita T., Goto Y., Tada K., Nagata N., Asano H., and Sugamura K. (1989). Monoclonal antibody defining molecule possibly identical to the p75 subunit of interleukin 2 receptor. J. Exp. Med. **169,** 1323–1332.

Tentori L., Longo L., Zuniga-Pflucker J.C., Wing C., and Kruisbeek A.M. (1988). Essential role of the interleukin 2-interleukin 2 receptor pathway in thymocyte maturation in vivo. J. Exp. Med. **168,** 1741–1747.

Toribio M.L., Alonso J.M., Barcena A., Gutierrez J.C., Hera A., Marcos M.A.R., Marquez C., and Martinez A.C. (1988). Human T-cell precursors: Involvement of the IL-2 pathway in the generation of mature T cells. Immunol. Rev. **104,** 55–79.

Uttenhove C., Coulie P.G., and Van Snick J. (1988). T cell growth and differentiation induced by interleukin-HP1/IL-6, the murine hybridoma plasmacytoma growth factor. J. Exp. Med. **167,** 1417–1427.

von Boehmer H., Crisanti A., Kisielow P., and Haas, W. (1985). Absence of growth by most receptor-expressing fetal thymocytes in the presence of interleukin-2. Nature **314,** 539–540.

